# Loss of Awareness or Urinary Dysfunction? Investigating Amyloidosis and Urinary Physiology in a Transgenic Mouse

**DOI:** 10.1093/geroni/igab046.2045

**Published:** 2021-12-17

**Authors:** Cara Hardy, Ramalakshmi Ramasamy, Dawn Rosenberg, Philip Scarpa, Xiangyou Hu, George Kuchel, Phillip Smith

**Affiliations:** 1 UConn Health, Farmington, Connecticut, United States; 2 University of Connecticut Health, Farmington, Connecticut, United States

## Abstract

Alzheimer’s disease (AD) is a devastating disorder primarily affecting older adults and is the most common neurodegenerative disease in the US. More than one in three AD patients experience AD-associated urinary dysfunction (ADUD), which directly contributes to their institutionalization. While ADUD has been clinically regarded as a result of poor cognitive control over urinary function, the physiology underlying loss of urinary control remains unknown. We hypothesize that amyloidosis in the CNS results in pathologic changes in urinary structure and function. Tg-APP/PS1DE9 mice were used before plaque deposition (4-6 months) and after plaque accumulation (8-10 months) and compared to WT littermates. Behavioral assays (open field testing and voiding spot assays) were performed to assess cortical function. Pressure-flow cystometry was conducted under urethane anesthesia to assess autonomic control of urinary function without cortical influence. Pharmacomyography of bladder strips was used to determine tissue-level changes in the absence of CNS input. In Tg-APP/PS1DE9 mice, plaque accumulation resulted in significant cystometric changes to voiding phase parameters, but not storage phase parameters. Pharmacologic studies showed decreased sensitivity to adrenergic stimulation without change in muscarinic sensitivity. Behavioral assays demonstrated significant differences between transgenic animals and WT in locomotion and voiding spot sizes. We interpret our data to support AD-related pathology of Aβ accumulation results in a distinct urinary phenotype in our model, analogous to the ADUD observed in AD patients. Establishing and verifying models of ADUD may improve the efficacy of treating ADUD and increase quality of life for patients and their caregivers.

